# Childhood cancers in a referral hospital in south-south Nigeria: a review of the spectrum and outcome of treatment

**DOI:** 10.11604/pamj.2015.22.325.6990

**Published:** 2015-12-02

**Authors:** Eno-Obong Edet Utuk, Enobong Emmanuel Ikpeme

**Affiliations:** 1 Department of Paediatrics, University of Uyo Teaching Hospital, Uyo, Akwa Ibom State, Nigeria

**Keywords:** Childhood, cancers, spectrum, outcome, Uyo, Nigeria

## Abstract

**Introduction:**

Childhood malignancies are now recognized as a growing global challenge, especially in resource poor settings. Although they constitute a smaller percentage of childhood illnesses in developing countries, compared with infectious diseases, the burden of cancer is still a tremendous problem on patients, families, the healthcare system, and the society. Data on the burden of childhood cancers across different regions is important, as there may be variations in incidences in different locations even within the same country. It will assist government agencies in better healthcare planning.

**Methods:**

An eight year retrospective analysis of all cancers diagnosed in children below the age of 18 years at the study centre between January 2007 and December 2014 was carried out. Case folders of all children diagnosed with malignancies within the study period were retrieved and analyzed with respect to age, gender, morphological or histological type of cancer, treatment modality, and outcome.

**Results:**

Eighty-four (84) children were diagnosed with various malignancies during the study period. Fourty-eight 48 (57.1%) were male and 36 (42.9%) were female giving a male to female ratio of 1.3:1. There were 27 cases (32.1%) of cancers recorded in children aged below 5 years and 35 cases (41.7%) were diagnosed in children between 5 to 10 years. Lymphomas were the most prevalent malignancies encountered accounting for 32 cases (38.1%). Burkitt's lymphoma constituted 22 (68.8%) of all lymphoma cases. The distribution of the four foremost malignancies recorded were as follows: Burkitt's lymphoma (22 cases; 26.2%), Nephroblastoma (12 cases; 14.3%), Rhabdomyosarcoma (6 cases; 7.1%) and 5 cases (6.0%) each Hodgkin's and non-hodgkin's lymphoma. Other malignancies included 4 cases (4.8%) each of acute leukaemia, neuroblastoma and retinoblastoma. There were three cases (3.6%) each of hepatoblastoma, and osteosarcoma among others. The cancer cure rate was very low 2.4%, losses to follow-up was 38.1% and 21.4% of patients died in the course of therapy either from advanced disease, complications of chemotherapy or late presentation.

**Conclusion:**

The distribution of the childhood malignancies in our environment shows similarity with reports from the same region and variation from other regions. The general outcome is very poor with a high percentage of discharge against medical advice and loss to follow up.

## Introduction

The burden of childhood cancers as a growing public health challenge is increasingly being recognized worldwide, including the developing nations [1]. Hitherto, communicable diseases and malnutrition had been the major causes of childhood illnesses and deaths, but with improving socioeconomic status, greater accessibility to healthcare services and improving immunization coverage and uptake, childhood non-communicable diseases are now recognized as an increasing significant challenge [1]. Tremendous progress has been made in the treatment and cure of childhood cancers in the last five decades. This is mostly due to advances in diagnosis and treatment, resulting in a cure or long term remission for a substantial number of children with cancer. It is however, pertinent to note that most of these improvements have occurred largely in developed countries. Caring for children with cancers pose a huge challenge on families and the healthcare system, especially in developing countries. This is so, because they often present too late for effective treatment, it usually portends prolonged hospital stay, varied invasive and non-invasive investigations for diagnosis, unavailability of hi-tech diagnostic facilities in many centers in resource-poor nations, therapeutic issues like unavailability of needed chemotherapy, and where available, high cost and non-affordability by most patients [2]. Developing countries contribute an estimated 60-80% of the total burden of all new cancer cases worldwide, and in these countries, about 60% or more of children with cancers die of the disease [2–4]. The age-standardized annual incidence usually ranges between 70 and 160 per million at age 0 to 14 years [5]. Some variation is seen between populations for some specific cancer types. Some of the largest variances are geographical and attributable to environmental factors, whereas ethnic variations may be a marker of genetic predisposition [6]. It is important for studies to be done on the spectra of childhood cancers in various localities. This gives valuable comparative data, and reveals the more common malignancies in an environment. It also forms the guiding framework for preventive and therapeutic interventions. This study, the first in our environment, was therefore carried out to document the spectrum of childhood cancers in the south-south region of Nigeria, and the outcome of treatment.

## Methods

This study is a retrospective analysis of all cases of cancer in children aged 0-18 years which were seen, diagnosed and managed in the Paediatric Haemato-oncology unit of the University of Uyo Teaching Hospital, Uyo over an eight year period from January 2007 to December 2014. Uyo is the capital city of Akwa Ibom State, situated in the South-South geo-political zone of Nigeria. It is located on the latitude 4'33 and 5'33 North and longitude 7'35 and 8'35, with abundant annual rainfall of 2095mm, mean daily temperature of 27^0^ Celsius and a relative humidity greater than 60%. It is within the tropical rain forest belt [7]. The University of Uyo Teaching Hospital is the only tertiary healthcare facility in Akwa Ibom State, located on the outskirts of Uyo about six kilometers from the centre of the city. The hospital is a three hundred and fifty-five bed health-care facility and serves as a referral centre, also accepting self reported cases. The Haemato-oncology unit of the department of Paediatrics sees, diagnoses and manages children with cancers. The materials used for the study included data obtained from the patients’ case folders, histopathology and bone marrow aspiration report forms. The records of all the patients were retrieved from the Records Department of the Hospital, and each of the cases were characterized with respect to age at diagnosis, gender, type of cancer, treatment modality and outcome. Routine histologic and/or histochemical procedures were employed in the processing of tissue biopsy or fine needle aspirate specimens for eventual histological diagnosis, while the peripheral blood and bone marrow samples in the cases of haematological malignancies were processed using standard techniques described by Dacie and Lewis [8]. For some cancers, immunologic or immuno-histochemical markers were employed in the process of diagnosis. Samples needing some special stains not available in this centre were sent to a referral centre (University College Hospital, Ibadan) for further evaluation. Other ancillary investigations which were carried out to help in diagnosis or management of the patients included X rays, serum urea, electrolytes and creatinine, and uric acid, full blood counts and platelets, erythrocyte sedimentation rate (ESR), liver and renal function tests. Cases which had incomplete documentation or missing histopathology /bone marrow report forms were excluded from the final analysis. The information retrieved from the above sources was recorded in pre-coded protocol forms designed for the study, and then entered into a study data base. The data was then analyzed using the Microsoft excel and results were presented in the form of frequency tables and percentages.

## Results

A total of eighty-four (84) cases of cancer were recorded in children aged 0-18 years between January 2007 and 31^st^ December 2014 at the University of Uyo Teaching Hospital (U.U.T.H.) giving an annual rate of 10.5 cases per annum. There were fourty-eight males (57.1%) and thirty-six females (42.9%) giving a male:female ratio of 1.3:1. The median age at diagnosis was 8 years (range 3 months to 17 years). The age and gender characteristics of the patients are shown in Table 1. The most prevalent malignancies encountered in our study were the lymphomas which accounted for 32 cases (38.1%). Burkitt's lymphoma was the commonest malignancy recorded (22 cases; 26.2%) and also the predominant lymphoma seen. Following Burkitt's lymphoma was Nephroblastoma (12 cases; 14.3%), Hodgkin's lymphoma (5 cases; 6.0%), Retinoblastoma (4 cases; 4.8%), Acute leukaemias (4 cases; 4.8%), and osteogenic sarcomas (3 cases; 3.6%). The distribution of the various malignancies is shown in Table 2. Burkitt's lymphoma was found to be commonest in the 5 < 10 year age group (22. 2%). Nephroblastoma and retinoblastoma were most prevalent in children below 5 years of age. Although Non Hodgkin's lymphoma was distributed in almost all the age groups, Hodgkin's lymphoma was encountered in children between the ages of 5 to 15 years. The age and gender distribution of the different cancers seen in patients were as outlined in Table 3. The methods used in diagnosis of patients were Fine needle aspiration cytology (FNAC) in 24 patients (28.6%), tissue biopsy in 49 cases (58.3%), Bone marrow aspiration (6 cases; 7.1%), and computerized tomography (CT) Scan in 5 patients (6.0%) Table 4. The treatment modalities for patients in this study are shown in Table 5. These were chemotherapy only in 52 patients (61.9%), Chemotherapy and surgery in 27 patients (32.1%). Patients evaluated and confirmed to need surgery with radiotherapy or a combination of chemotherapy, surgery and radiotherapy were all referred to other centres with such facilities and these constituted five (6.0%) of the patients. All the patients in addition to the above treatment had supportive therapy in the form of intravenous fluids, transfusion of blood and blood products, analgesics and counselling. The treatment outcome is as shown in Table 6.

**Table 1:** Age and gender distribution of patients, with year of diagnosis

Gender	N (%)
Males	48 (57.1%)
Females	36 (42.9%)
**Total**	84 (100.0%)
**Age group**	
<1 year	3 (3.6%)
1-<5 years	24 (28.6%)
5-<10 years	35 (41.6%)
10-<15years	17 (20.2%)
≥15years	5 (6.0)
**Total**	84 (100.0)
**Year of Diagnosis**	
2007	9 (10.7%)
2008	16 (19.1%)
2009	19 (22.6%)
2010	13 (15.5%)
2011	9 (10.7%)
2012	6 (7.1%)
2013	5 (6.0%)
2014	7 (8.3%)
**Total**	84 (100.0)

**Table 2:** Frequency of the various cancers seen in the patients

Diagnosis	N (%)
Burkitt's lymphoma	22 (26.2%)
Nephroblastoma	12 (14.3%)
Rhabdomyosarcoma	6 (7.1%)
Hodgkin's lymphoma	5 (6.0%)
Non-hodgkin's lymphoma	5 (6.0%)
Neuroblastoma	4 (4.8%)
Leukaemia	4 (4.8%)
Retinoblastoma	4 (4.8%)
Hepatoblastoma	3 (3.6%)
Osteosarcoma	3 (3.6%)
Naso-pharyngeal carcinoma	2 (2.4%)
Histiocytosis	2 (2.3%)
CNS tumours	2 (2.3%)
Neurofibrosarcoma	1 (1.2%)
Mucoepidermoid carcinoma	1 (1.2%)
Unspecified	8 (9.5%)
**Total**	84 (100.0)

**Table 3:** Age and gender distribution of the different cancers seen in patients

Type of cancer	<5		5- < 10		10 < 15		≥15		Total		Grand total
	M	F	M	F	M	F	M	F	M	F	
Burkitt's lymphoma	2	3	5	10	2	0	0	0	9	13	22
Nephroblastoma	4	3	3	2	0	0	0	0	7	5	12
Rhabdomyosarcoma	1	1	1	0	2	1	0	0	4	2	6
Hodgkin's lymphoma	0	0	2	0	2	1	0	0	4	1	5
Non-hodgkin's lymphoma	0	1	2	1	1	0	0	0	3	2	5
Neuroblastoma	1	1	0	1	0	1	0	0	1	3	4
Leukaemia	1	1	0	1	0	0	0	1	1	3	4
Retinoblastoma	1	1	1	1	0	0	0	0	2	2	4
Hepatoblastoma	2	1	0	0	0	0	0	0	2	1	3
Osteosarcoma	0	0	0	1	2	0	0	0	2	1	3
Naso-pharyngeal carcinoma	0	0	0	0	2	0	0	0	2	0	2
Histiocytosis	0	0	2	0	0	0	0	0	2	0	2
CNS Tumours	0	0	0	2	0	0	0	0	0	2	2
Neurofibrosarcoma	0	0	0	0	1	0	0	0	1	0	1
Mucoepidermoid tumour	0	0	0	0	1	0	0	0	1	0	1
Unspecified	3	0	0	0	1	0	3	1	7	1	8

**Table 4:** Method of diagnosis of the cancers

Method of diagnosis	N(%)
*FNAC	24(28.6)
Tissue biopsy	49(58.3)
Bone marrow aspiration	6(7.1)
^×^CT scan	5(6.0)
Total	84(100.0)

* Fine needle aspiration cytology;

× Computed tomography scan

**Table 5:** Mode of treatment of the cancers

Mode of treatment	N(%)
Chemotherapy only	52(61.9)
Surgery + chemotherapy	27(32.1)
Surgery ± radiotherapy	3(3.6)
Surgery±chemotherapy± radiotherapy	2 (2.4)
Total	84(100.0)

**Table 6:** Outcome of treatment

Outcome	N(%)
Cure	2(2.4)
Remission	2(2.4)
Discharge against medical advice	25(29.7)
Referred	5(6.0)
Loss to follow-up	32(38.1)
Death	18(21.4)
**Total**	84(100.0)

## Discussion

The study recorded a total of 84 childhood cancers over a period of eight years, with an annual average of 10.4 cases per annum. This is much lower than the 23.0 and 17.4 cases per year respectively reported from two different studies in Jos, northern Nigeria [9, 10], but comparable to the 12 cases per annum report from south-eastern and south-south Nigeria [11, 12]. It is higher than the 7 and 3 cases annual rates respectively seen by Agboola et al from Sagamu, south-western Nigeria [13] and Onwasigwe in Enugu, eastern Nigeria [14]. These figures indicate the variations in incidences that can occur even within the same country, and may be attributed to the different geographical characteristics, level of patient inflow and patronage of the various health institutions where these studies took place. The slight male preponderance noticed in this study is comparable to reports from other centres in Nigeria [9–13]. Soyemi et al [15] however found an equal male to female ratio from their study in Lagos, western Nigeria. Burkitt's lymphoma was the commonest childhood malignancy encountered in this study. This is similar to reports from other centres in Nigeria [2, 9, 12, 16, 17]. However, it differs from the reports by Tanko et al [10] in Jos, north central Nigeria who found rhabdomyosarcoma as the commonest childhood solid tumour in their study between 2002 -2006 [10]. The observed difference in results from a later study [9] in the same centre may be attributed to the difference in methodology. Tanko et al [10] conducted their study only on solid surgical specimens obtained from the histopathology laboratory. This would greatly affect their results and the cancer pattern documented as cancers diagnosed by other methods such as fine needle aspiration cytology and imaging studies would be excluded. Another study from Kano, northern Nigeria [18] recorded retinoblastoma as the commonest childhood malignancy followed by Burkitt's lymphoma. The three most frequent cancers seen in this study were very similar to observations in Calabar, also located in the same region as this study site, with similar geographical climatic conditions. The observed slight differences in the spectrum of childhood cancers between these northern and southern parts of Nigeria may therefore be as a result of climatic differences, or genetics and different levels of exposure to environmental toxins in these areas. Soyemi et al [15] in Lagos, found a higher incidence of nephroblastoma, but in that study, children with lymphomas and leukaemias were excluded and only those with mesenchymal type of tumours were assessed, thus may not be a true reflection of the overall picture.

This study found a commoner frequency of Burkitt's lymphoma in females than males. This finding is in contrast with observations from most studies done in Nigeria, which had a higher frequency in males [9, 18]. The reason for this is not very clear. The decline in the cases of Burkitt's lymphoma observed in this study was likewise reported by Ojesina et al [19] in Ibadan, western Nigeria. Burkitt's lymphoma is known to be endemic in tropical Africa, and its area of endemicity is identical with that of malaria. An interplay between malaria, Ebstein Barr Virus and malnutrition has been the favoured hypothesis in its aetiology [20]. It is likely, that with the improved and widespread knowledge and use of the national antimalarial treatment with the artemisinin combination drugs even over the counter, the subsequent decline in clinical malaria, has had a positive role in the observed declining incidence [4, 6]. Nephroblastoma was the next common malignancy, comparable to documentation from Omotayo et al in Ilorin, western Nigeria, and Ekanem et al [12] in Calabar, but slightly different from other studies [10, 17] which recorded retinoblastoma and rhabdomyosarcoma as commonest. The commonest method of diagnosis used was the Fine Needle Aspiration Cytology (FNAC) as also documented in other studies [2, 9]. The less invasiveness, simplicity, safety and comparable diagnostic accuracy of the procedure make its use favourable in most cases of solid malignancies [21]. The commonest treatment modality was ‘chemotherapy only'in 61.9% of our patients. About 21.4% of patients had surgery in addition to chemotherapy. Few other cases needing mainly radiotherapy and/or specialized surgery before chemotherapy, such as the few children with intracranial tumours, muco-epidermoid carcinoma and neurofibrosarcoma respectively were referred to other tertiary centres after preliminary clinical/radiological diagnosis, as our centre does not have a neurosurgical unit or radiotherapy unit presently. A child with nephroblastoma was also referred out based on parents’ wish to receive treatment from another tertiary centre closer to the financial sponsors of child's treatment.

Most patients received one form of supportive care or another during the course of treatment. Major challenges encountered in the management of patients included unavailability of some needed cytotoxic drugs, high cost where available and the unwillingness on the part of some parents / caregivers to continue spending indefinitely after prolonged stay in the hospital, coupled with no remarkable clinical changes, especially in those who presented with advanced stages of the disease. This was a similar experience in many centres in the country [2, 9, 12, 22]. The high default rate and marked loss to follow-up noted may be because in most developing countries, cancer is still a terror to the child and family, and in many communities, still being viewed as not curable, and a disease that cannot be overcome using orthodox methods. Rather, traditional healers, spiritual healers including churches are highly patronized for respite, especially in this study locality [20, 23]. This may be highly accountable to many of the patients who discharged against medical advice, and were lost to follow-up [20, 24, 25]. It can be extrapolated that most of this children died at home, and such deaths are ‘invisible’, as there exists no records. During treatment and afterwards, parents and relations often suffer from depression and burn-out. They therefore need to be supported by trained psychologists and support groups during this period. Where these are lacking, they eventually succumb to negative influences in the community. The study centre was initially run as a federal medical centre, but with its upgraded status to a Teaching Hospital, more specialized and dedicated services are now incorporated to cater for these children with cancers. The follow-up status of children with cancers is now improved. Two children, who presented early with nephroblastoma and followed through with treatment and a five year follow-up, have no residual signs of any disease, as well as two children with retinoblastoma who are in remission. Specific blood products such as granulocyte transfusions were not readily available during cycles of chemotherapy for children needing such, which constituted limitations of the study. This necessitated fresh whole blood being used in most cases and this negates the principle of rational use of blood and blood products.

## Conclusion

The distribution of the childhood malignancies in our environment shows similarity with reports from the same region and variation from other regions. The general outcome is very poor with a high percentage of discharge against medical advice and loss to follow up. We therefore recommend large scale community awareness on cancers and its prognosis with greater resource allocation for its treatment. Also, training of personnel and support staff is pertinent. This will aid our fight in the early detection, treatment and prevention of childhood cancers.
